# Anti-Atopic Dermatitis Effect of *Seaweed Fulvescens* Extract via Inhibiting the STAT1 Pathway

**DOI:** 10.1155/2019/3760934

**Published:** 2019-03-17

**Authors:** Tae-Young Gil, Yun-Mi Kang, Ye-Jin Eom, Chul-Hee Hong, Hyo-Jin An

**Affiliations:** ^1^Department of Pharmacology, College of Korean Medicine, Sangji University, Wonju-si, Gangwon-do 26339, Republic of Korea; ^2^Department of Korean Medicine Ophthalmology & Otolaryngology & Dermatology, College of Korean Medicine, Sangji University, Wonju-si, Gangwon-do 26339, Republic of Korea

## Abstract

*Seaweed fulvescens* (SF) is a green alga rich in chlorophyll with unique flavor and taste. It is also called Maesaengi which has antioxidant and other physiological activities. In the present study, we evaluated the therapeutic effects of SF in a mouse model of *Dermatophagoides farinae* body-induced atopic dermatitis (AD) and in tumor necrosis factor-*α* and interferon-*γ*-stimulated HaCaT keratinocytes. SF treatment (200 mg/mouse) inhibited the development of AD symptoms, compared to that in the control group, as evidenced from the improved dorsal skin lesion, reduced thickness and infiltration of inflammatory cells and smaller lymph nodes, and reduced levels of proinflammatory cytokines. In HaCaT keratinocytes, SF (10, 25, and 50 *μ*g/mL) suppressed the production of proinflammatory cytokines in a dose-dependent manner. In addition, SF reduced the phosphorylation of signal transducer and activator of transcription 1, which is one of the major signaling molecules involved in cellular inflammation. These results suggested that SF could be a potential therapeutic alternative for the treatment of AD.

## 1. Introduction

Atopic dermatitis (AD) is an eczematous, frequently pruritic chronic inflammatory skin disease [1]. Although the pathogenesis of AD is not fully elucidated, various studies have shown that AD pathogenesis is related to various factors, including allergies, genetic factors, environmental factors, and immunological functions [2]. AD is characterized by some typical symptoms, such as itchiness, skin redness, increase in skin thickness, and skin dryness caused by inflammatory responses [3]. It is mediated by the infiltration of inflammatory cells including macrophages, mast cells, and eosinophils [4].

The inbred NC/Nga mouse strain was established in 1957 using a Japanese mouse strain (Nishiki-Nezumi). NC/Nga mice are widely using as an animal model for the investigation of the pathogenesis of human AD. Patients with AD are highly sensitive to house dust mite allergens. One of the most common mites in house dust, *Dermatophagoides farinae* (Df), is a well-known major environmental allergen [5]. It was reported that Df extract could induce AD-like clinical skin lesions in NC/Nga mice [6].

Keratinocytes, which constitute the majority of the epidermal cells, play a critical role in the pathogenesis of inflammatory skin disorders, including AD [7]. Keratinocytes produce different cytokines and chemokines in response to stimulation by tumor necrosis factor-alpha (TNF-*α*) and interferon-gamma (IFN-*γ*) [8]. Various cytokines and chemokines are regarded as important regulators of the pathogenesis of AD. In addition, stimulation of keratinocytes by TNF-*α* and IFN-*γ* can lead to the activation of different signaling pathways such as nuclear factor-kappa B, mitogen-activated protein kinases, and signal transducer and activator of transcription (STAT) 1 pathways [9].

*Seaweed fulvescens* is a filamentous chlorophyll alga that is found in the North Atlantic and Northern Pacific regions, including South Korea and Japan [10–12]. It has been consumed as a unique food flavor with soft texture to treat stomach disorders and hangovers in the Southwestern province of Korea [13]. Several studies showed that SF extracts reduced cholesterol levels in hypercholesterolemic rats [14], inhibited melanogenesis [15], and induced apoptosis in AGS gastric cancer cells [16]. In addition, it was shown to possess antioxidant and other physiological activities [17]. Furthermore, there are many studies that analyzed the compounds of SF [18]. Chlorophyll, the major colored compound in SF, was shown to exhibit anti-inflammatory effects [19]; thus, it could be beneficial in inflammatory diseases, such as AD. Moreover, many cosmetic products contain chlorophyll owing to its effects on the skin, including antiwrinkle activity [20]. Fucoidan, a fucose-rich polysaccharide, is another compound present in SF. It can be extracted from seaweeds and has diverse biological properties such as anticancer, wound healing, and tissue engineering activities [21]. However, the effects of SF in AD have not been investigated yet. In this context, we investigated the effects of SF in AD since it is one of the complex skin diseases resulting from disrupted skin immunity. In this study, we examined whether SF could alleviate AD in Df-induced AD mice and in TNF-*α*- and IFN-*γ*-stimulated HaCaT keratinocytes.

## 2. Materials and Methods

### 2.1. Chemicals and Reagents

MTT and Griess reagents were imported from Sigma Chemical Co. (St. Louis, MO, USA). Dulbecco's modified Eagle's medium (DMEM), fetal bovine serum (FBS), penicillin, and streptomycin were got from Life Technologies Inc. (Grand Island, NY, USA). Dimethylsulfoxide (DMSO) was bought from Junsei Chemical Co. Ltd. (Tokyo, Japan). The enzyme immunoassay (EIA) kits for TNF-*α* and IL-6 were got from R&D System (Minneapolis, MN, USA). SYBR Premix Ex Taq was bought from Takara (Shuzo, Shiga, Japan). TNF-*α*, IL-6, and GAPDH oligonucleotide primers were bought from Bioneer (Daejeon, Chungbuk, South Korea). Primary antibodies against p-STAT1 (Ser727) and STAT1 were obtained from Cell Signaling Technology (Danvers, MA, USA). Primary antibodies against p-STAT1 (Tyr701) and *β*-actin as well as peroxidase-conjugated secondary antibodies were got from Santa Cruz Biotechnology Inc. (Santa Cruz, CA, USA).

### 2.2. Preparation of SF Extract

SF was purchased from Haedream Inc. (Hwasun-gun, Jeollanam-do, South Korea). It was harvested from Jeongnamjin, Jangheung-gun, Jeollanam-do, freeze-dried, and stored at -70°C. The sample (100 g) was extracted with 1 L of 70% aqueous EtOH for 4h. After decoction, it was concentrated using a rotary vacuum evaporator (EYELA, Tokyo, Japan). Then, the sample was freeze-dried using a freeze dryer (FDU-1200, EYELA, Tokyo, Japan) and kept at -80°C until analysis.

### 2.3. Cell Culture and Sample Treatment

Human adult, low-calcium, high-temperature (HaCaT) keratinocytes were kindly provided by Prof. Kyung-Tae Lee (Kyung Hee University, South Korea) and cultured at 37°C in DMEM supplemented with 10% FBS, penicillin (100 U/mL), and streptomycin (100 *μ*g/mL) under a humidified atmosphere with 5% CO_2_. Cells were treated with SF at concentrations of 10, 25, and 50 *μ*g/mL. Then, they were stimulated with TNF-*α* and IFN-*γ* mixture (10 ng/mL, each) for the indicated time.

### 2.4. Cell Viability Assay

Cell viability was determined using the colorimetric MTT assay. Briefly, SF-treated cells were incubated for 24h. Next, the cells were kept in 5 mg/mL MTT solution for 4 h at 37°C. DMSO was used to dissolve the insoluble formazan product after removal of the supernatant. Cell viability was measured at 570 nm using an Epoch microplate spectrometer (BioTek, Winooski, VT, USA). Experiments were conducted in thrice in parallel for each concentration of SF, and the results were expressed as the mean ± standard deviation (SD).

### 2.5. Experimental Animals and Sample Treatment

**Figure 1:**
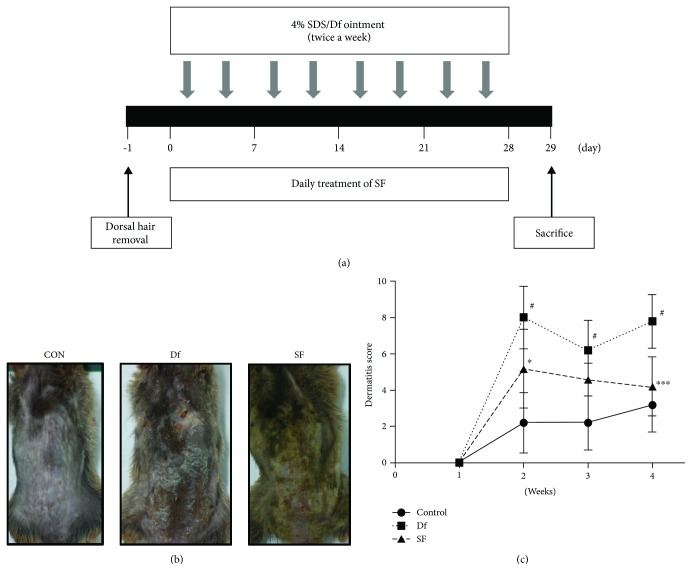
Effects of SF on Df-induced AD-like skin lesions in NC/Nga mice. (a) Schematic depiction of the development of SDS/Df-induced AD and treatment with SF. (b) Representative photographs of the dorsal regions of mice from each group at 29 days after AD induction and treatment with SF. CON: control mouse group, Df: Df-induced atopic dermatitis (AD) NC/Nga mice, and SF: *seaweed fulvescens* (SF) extract-treated AD mice. (c) Dermatitis scores for 4 weeks. Dermatitis score were determined as the sum of scores graded as 0 (none), 1 (mild), 2 (moderate), or 3 (severe) for each of the four symptoms (erythema/hemorrhage, scarring/dryness, edema, and excoriation/erosion). Data are expressed as the mean ± standard deviation (SD; *n* = 6). Data were analyzed using one-way analysis of variance followed by Dunnett's post hoc test. ^∗^ *P* < 0.05 and ^∗∗∗^ *P* < 0.001 versus the Df group; ^#^ *P* < 0.05 versus the control group.

NC/Nga male mice (20-25 g body weight, 8 weeks old) were obtained from Daehan Biolink Co. (Daejeon, Republic of Korea). Animals were kept under standard conditions according to the guidelines for laboratory animal care and usage. The guidelines were adopted and promulgated by Sangji University in accordance with the requirements of the National Institutes of Health. Prior to the experiments, the Institutional Animal Care and Use Committee (IACUC) of Sangji University approved all the experimental protocols (IACUC animal approval protocol No. 2018-20). Mice were housed (6 mice/cage), acclimatized to the animal room, and fed with standard laboratory chow. They were maintained under constant conditions of 12 h dark/light cycles, temperature of 20 ± 5°C, and humidity of 40-60% for a week. After acclimatization for a week, the mice were randomly divided into three groups. To induce AD-like skin lesions, the back skin was topically treated with 150 *μ*L of 10 mg/mL crude extract of Df (Biostir® AD; Biostir, Hyogo, Japan). Mite antigen was applied repeatedly twice weekly for 4 weeks (Figure 1(a)). The skin barrier was disrupted with 150 *μ*L of 4% SDS following the application of Df ointment and SF for 3 h. Df-induced dermatitis mice were divided into three groups: (1) the control group with no SDS and Df application; (2) the Df-treated group with Df application (Df-induced AD-like lesion); and (3) the SF-treated group (200 mg/mouse) with Df application for 4 weeks. Mice were sacrificed at 4 weeks after the first application of Df, and blood was collected from the orbital sinus. The dorsal skin tissues were isolated for histological examination.

### 2.6. Evaluation of Dermatitis Severity

Severity of eczema was scored using the Merkmal of symptom score, as described by Sone and colleagues [22]. The deterioration of dermatitis was evaluated once weekly. The aggravation of edema, scarring/dryness, erythema/hemorrhage, and excoriation/erosion was scored as follows: 0, none; 1, mild (severity < 20%); 2, moderate (severity = 20-60%); and 3, severe (severity > 60%). The total of the individual points was used as the dermatitis score.

### 2.7. Histological Analysis of Skin Lesions

Skin samples of the dorsal area were isolated after euthanasia. The samples were fixed in 10% buffered formalin and then embedded in paraffin. Then, they were sectioned into 8 *μ*m slices and were stained with H&E. Pathological changes, such as hyperkeratosis, dermal edema, epidermal and dermal hyperplasia, vesicular formation, parakeratosis, and inflammation, were evaluated. Selected sections were stained with toluidine blue for the assessment of mast cell infiltration. The average count of mast cells in each specimen was used to determine mast cell density/mm^2^. Images were captured under an optical microscope (Leica, Wetzlar, Germany) using Leica software.

### 2.8. Cytokine Assays

Blood was collected from each mouse orbital sinus at the end of the experiment. Serum was obtained by centrifugation at 1700 × *g* for 30 min and kept at -70°C until analysis. The serum levels of total TNF-*α* and IL-6 were measured using mouse TNF-*α* and IL-6 ELISA kits (BD OptEIA TM, BD Science, CA, USA), according to the manufacturer's instructions. Culture media were obtained approximately 24 h after treatment with SF and stored at -70°C. The production levels of TNF-*α* and IL-6 were assessed using EIA kits for human (BD OptEIA TM, BD Science, CA, USA) according to the manufacturer's instructions.

### 2.9. Measurement of Relative mRNA Expression Level Using qRT-PCR

Total RNA was isolated from the back skin tissue using an RNeasy fibrous tissue mini kit (Qiagen, Valencia, CA), according to the manufacturer's instructions. cDNA was obtained using isolated total RNA (2 *μ*g), d(T)16 primer, and AMV reverse transcriptase. Relative gene expression was quantified by quantitative RT-PCR (Real Time PCR System 7500, Applied Biosystems, Foster City, CA, USA) with SYBR premix Ex Taq. The synthesized cDNAs had a size of 200 bp. Results were expressed as the elative optical density to that of *GAPDH*. The primer sequences are summarized in Table 1.

### 2.10. Western Blot Analysis

Protein extracts were prepared using PRO-PREP™ protein extraction solution (Intron Biotechnology, Seoul, South Korea) and homogenized at 4°C. Tissue debris of the supernatant was removed with microcentrifugation followed by immediate freezing. The protein concentration was determined using the Bio-Rad protein assay reagent, according to the manufacturer's instructions. After 10-12% SDS-polyacrylamide gel electrophoresis, protein from each group was electro-blotted onto a polyvinylidene difluoride (PVDF) membrane. The immunoblot was incubated with a blocking solution (2.5-5% skim milk) for 30 min at room temperature and subsequently incubated overnight with a primary antibody (dilution, 1 : 1000 in Tween 20/Tris-buffered saline [TBST]) at 4°C. After washing thrice with TBST, blots were incubated with a horseradish peroxidase-conjugated secondary antibody (dilution, 1 : 2000) for 2 h at room temperature. Blots were washed again thrice with TBST and then visualized using enhanced chemiluminescence (ECL; GE healthcare, WI, USA). Densitometric analysis was performed using Bio-Rad Quantity One software. In addition, other immunodetected bands responded to ECL solution (Ab signal, Seoul, South Korea) and were displayed on an X-ray film (Agfa, Belgium).

### 2.11. Statistical Analysis

Data were expressed as the mean ± SD of three experiments. Comparisons among groups were carried out using one-way analysis of variance (ANOVA) followed by Dunnett's post hoc test. *P* values < 0.05 were considered statistically significant.

## 3. Results

### 3.1. Effects of SF on Df-Induced AD-Like Skin Lesions in NC/Nga Mice

Pruritus, eczematous, and inflammatory skin lesions are usual symptoms in NC/Nga mice with AD. To induce house dust mite antigen-induced AD in NC/Nga mice, Df ointment was applied repeatedly to the skin lesion twice weekly for 4 weeks [23] (Figure 1(a)). As shown in Figure 1(b), repeated application of Df ointment to NC/Nga mice induced skin dryness, followed by erythema, hemorrhage, and edema. Eventually, the skin became thick. In addition, severe erythema, hemorrhage, edema, scarring, erosion, and excoriation occurred. However, treatment with SF reduced these skin symptoms. Using the Merkmal of symptom score, skin conditions were evaluated once weekly after the second application of Df for 28days (Figure 1(c)).

### 3.2. Effects of SF on Skin Integrity and Mast Cell Infiltration in Df-Treated NC/Nga Mice

**Figure 2:**
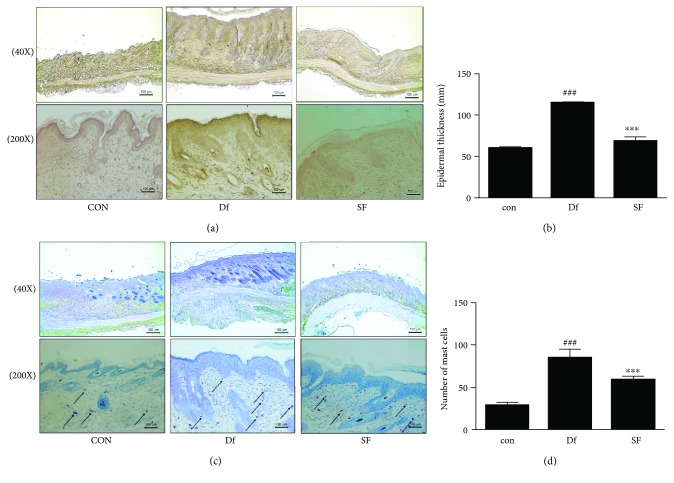
Effects of SF on skin integrity and mast cell infiltration in Df-treated NC/Nga mice. (a) Histological examination of NC/Nga mice. The tissues were excised and fixed in 10% formaldehyde. Then, they were embedded in paraffin and sectioned. The sections were stained with H&E (40x and 200x magnifications). (b) Epidermal thickness was examined after sacrifice. (c) Staining with toluidine blue was used to identify mast cells, and (d) mast cell counts were determined using a microscope at 200x magnification. Data were expressed as the mean ± SD (*n* = 6). Data were analyzed using one-way analysis of variance followed by Dunnett's post hoc test. ^∗∗∗^ *P* < 0.001 versus the Df group; ^###^ *P* < 0.001 versus the control group.

To determine whether SF treatment reduced Df-induced inflammation and immune-cell infiltration in AD-like skin lesion, histological analysis was performed. Hematoxylin and eosin (H&E) staining showed epithermal hyperplasia, edema, and accumulation of inflammatory cells in the epidermis of the Df-treated group, compared to those in the control group. However, treatment with SF reduced these inflammatory responses (Figures 2(a) and 2(b)). To investigate inflammatory cell infiltration into the skin after Df application, mast cells in skin tissue sections were marked with toluidine blue staining. The number of mast cells in the dermis significantly increased in the Df-treated group, compared to that in the control group (Figures 2(c) and 2(d)).

### 3.3. Effects of SF on Df-Induced Systemic Immunological Abnormalities in NC/Nga Mice

**Figure 3:**
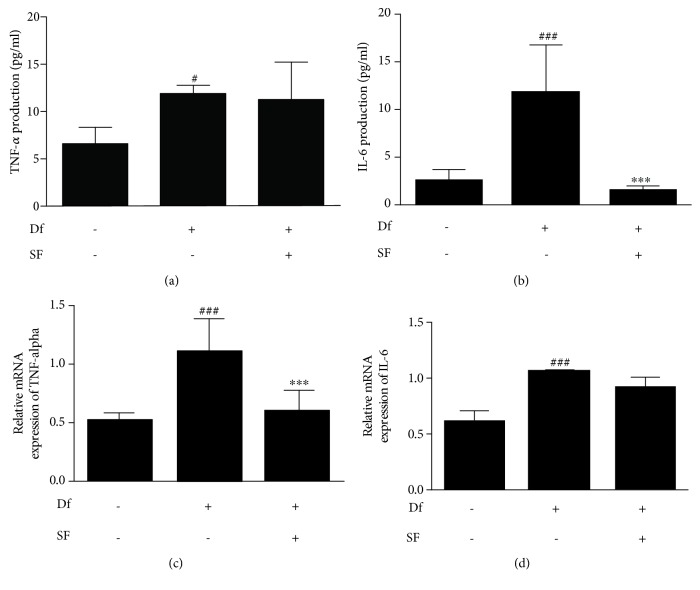
Effects of SF on Df-induced systemic immunological abnormalities in NC/Nga mice. (a–d) The mRNA expression and production of inflammatory cytokines were determined in skin lesions. Total RNA was isolated from the skin lesions of NC/Nga mice treated with SF, and quantitative reverse transcription polymerase chain reaction was performed. The production of inflammatory cytokines was measured by ELISA. Data were presented as the mean ± SD (*n* = 6). Data were analyzed using one-way analysis of variance followed by Dunnett's post hoc test. ^∗^ *P* < 0.05 and ^∗∗∗^ *P* < 0.001 versus the Df group; ^#^ *P* < 0.05, ^##^ *P* < 0.01, and ^###^ *P* < 0.001 versus the control group.

Since AD frequently develops as a systemic disease [24], we investigated whether application of SF could affect systemic immune responses. Mice in the Df-treated group had larger and heavier lymph nodes than those in the Df-untreated control group. Treatment of SF had no effect on lymph nodes weight. Furthermore, spleen weight showed little differences among groups (Supplementary Figures 1(a) and 1(b)). The analysis of the gene expression and production of inflammatory cytokines showed that SF affected Df-induced systemic immune responses (Figures 3(a)–3(d)). Proinflammatory cytokines, such as TNF-*α* and IL-6, increased in Df-induced NC/Nga mice. However, SF reduced their skin mRNA expression and serum levels.

### 3.4. Effect of SF on the Phosphorylation of STAT1 in NC/Nga Mice

**Figure 4:**
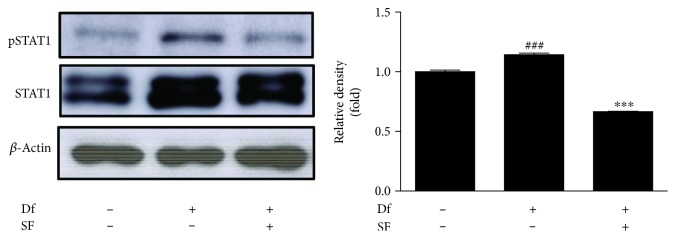
Effects of SF on inflammatory signaling pathways in Df-induced skin lesions. Protein was isolated from normal or Df-induced dermatitis dorsal skin. Phosphorylation of STAT1 was measured by western blot analysis. The graph shows the ratio of phosphorylated STAT1 to total STAT1. Values represent the mean ± SD of triplicate independent experiments. ^∗∗∗^ *P* < 0.001 versus the Df group; ^###^ *P* < 0.001 versus the control group.

STATs are one of the families of nuclear proteins that mediate the action of various cytokines, such as interleukins and interferons [25]. Inhibiting the activation of STAT1 is regarded as an important step to treat skin inflammatory diseases [26]. Therefore, we evaluated the effects of SF on the phosphorylation of STAT1 in AD-like dermatitis skin lesions in NC/Nga mice. As shown in Figure 4, SF administration suppressed the phosphorylation of STAT1 in the dorsal skin compared to that in the Df-treated group.

### 3.5. Effects of SF on the mRNA Expression and Production of Inflammatory Mediators in HaCaT Keratinocytes

**Figure 5:**
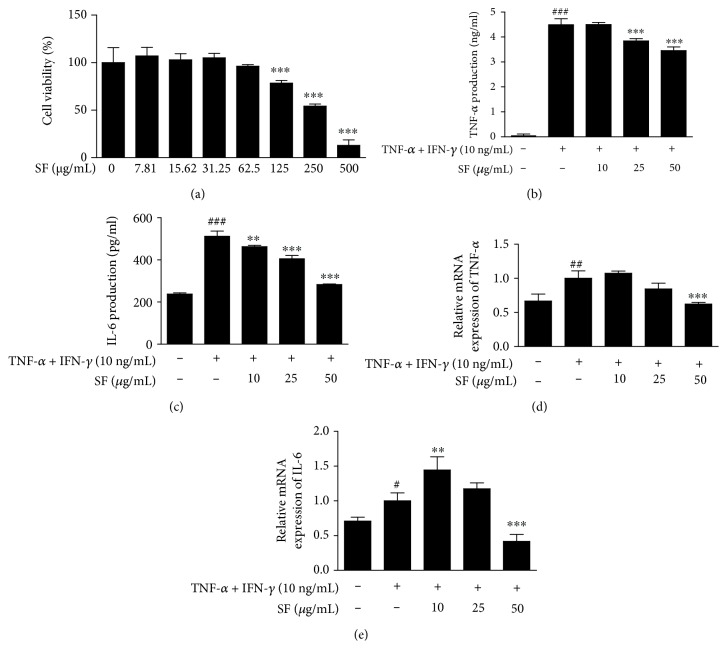
Effects of SF on the mRNA expression and production of inflammatory cytokines in HaCaT keratinocytes. (a) Cell viability was determined using the 3-(4,5-dimethyl-2-thiazolyl)-2,5-diphenyl-2H-tetrazolium bromide (MTT) assay. Cells were seeded in 96-well microplates at 1 × 10^5^ cells/well, and various concentrations of SF were added to each well for 24 h. The mRNA expression levels (b, d) and production of inflammatory cytokines (c, e) were determined in HaCaT keratinocytes. Total RNA was isolated from the cells treated with SF for 6 h, and quantitative reverse transcription-polymerase chain reaction was performed. The production of inflammatory cytokines was measured by ELISA. Proinflammatory cytokine levels were measured in the culture supernatants from cells treated with SF (10, 25, and 50 *μ*g/mL) and TNF-*α* and IFN-*γ* (each 10 ng/mL) for 24 h. Values represent the mean ± SD of three independent experiments. ^∗∗^ *P* < 0.01 and ^∗∗∗^ *P* < 0.001 versus the Df group; ^#^ *P* < 0.05, ^##^ *P* < 0.01, and ^###^ *P* < 0.001 versus the control group.

Epidermal keratinocytes play multiple roles in the immune responses related to AD and other skin diseases [27]. First, the effect of SF on cell viability was examined using the MTT assay. SF treatment at 7.8125-125 *μ*g/mL did not show cytotoxicity in HaCaT keratinocytes (Figure 5(a)). Therefore, further experiments were conducted at concentrations of 10, 25, and 50 *μ*g/mL. To investigate whether SF could downregulate the expression of TNF-*α*/IFN-*γ*-induced proinflammatory cytokines, we carried out quantitative reverse transcription polymerase chain reaction (qRT-PCR) and ELISA. The expression levels of proinflammatory cytokines were reduced by SF treatment in a dose-dependent manner. In addition, SF downregulated the production of IL-6 and TNF-*α* in stimulated HaCaT cells (Figures 5(b)–5(e)).

### 3.6. Effect of SF on the Phosphorylation of STAT1 in HaCaT Keratinocytes

**Figure 6:**
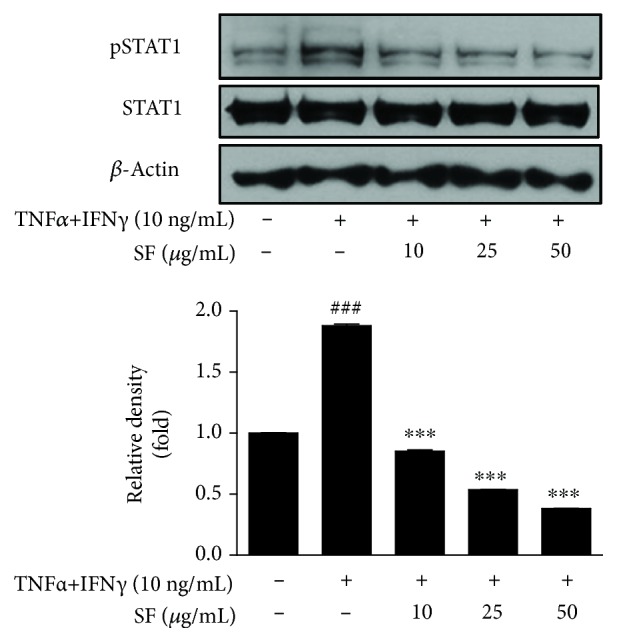
Effect of SF on the activation of STAT1 in HaCaT keratinocytes. Phosphorylation of STAT1 was measured in HaCaT cells pretreated with SF (10, 25, and 50 *μ*g/mL) for 1 h and stimulated with TNF-*α* and IFN-*γ* (10 ng/mL each) for 2 h. The graph shows the ratio of phosphorylated STAT1 to total STAT1. Values represent the mean ± SD of three independent experiments. ^∗∗∗^ *P* < 0.001 versus the Df group; ^###^ *P* < 0.001 versus the control group.

The intracellular mechanisms underlying the secretion of various inflammatory mediators involve STAT1 as a crucial molecule among the IFN-*γ*/cytokine signaling pathways [28]. Here, we examined the effect of SF on pSTAT1 and STAT1 expression induced by TNF-*α*/IFN-*γ* mixture. Results suggested that SF reduced the activation of STAT1 in HaCaT cells (Figure 6).

## 4. Discussion

AD is a recurring inflammatory and itchy skin disorder. Its clinical symptoms include skin dryness, erythema, oozing and crusting, and lichenification [29]. Severe pruritus is a characteristic of the disease and is the reason for much of the disease burden for patients and their families. General standard treatments, such as topical steroids and calcineurin inhibitors, are used for the relief of severe AD. However, the potential adverse effects limit their use; therefore, the development of new treatments with good efficacy and few side effects is required [30].

Several studies have shown that marine plants have pharmaceutical and biological activities [31–34]. SF, a marine plant, has been shown to possess various biological properties, such as antioxidant, antihypercholesterolemic, and melanogenesis inhibitory effects [14–17]. However, the biological effect of SF extract remains unclear. Therefore, this study investigated the anti-AD effects of SF *in vivo* and *in vitro*.

The pathogenesis of AD involves not only environmental, genetic, and psychological factors but also immune system dysfunction and epidermal barrier defects [35]. Environmental exposures can occur through the respiratory route and might result in the induction or exacerbation of dermatitis in patients with AD [36]. House dust mite is the most common aeroallergen in Asian countries [37]. Common species of house dust mites include *Dermatophagoides pteronyssinus* (Der p), Df, and *Blomia tropicalis* (BT) [38]. In this study, the antiallergic effects of SF were examined following the application of a mite antigen Df ointment to NC/Nga mice. Although the severity of AD in humans could be different from that in the mouse model, the clinical features and symptoms occurring in NC/Nga mice were very similar to those occurring in patients with AD [5]. Treatment of the dorsal skin with SF reduced skin lesion (Figure 1(b)) and dermatitis score (Figure 1(c)). Epidermal thickening and infiltration of mast cells in the epidermis/dermis were observed in the Df-treated mice (Figures 2(a)–2(d)). These results showed that SF inhibited Df-induced AD-like skin symptoms in the NC/Nga mouse model.

AD is a skin allergic disease, resulting from dermal inflammation. The loss of the immunological balance between Th1 and Th2 cells is one of major pathogenic events occurring in AD [39]. Acute AD skin lesions exhibit Th2-dominant responses characterized by dermal infiltration of CD4^+^ or CD8^+^ T cells and eosinophils, as well as upregulated skin expression of Th2 cytokines [40]. However, Th1 responses are characterized by the production of effector cytokines, such as TNF-*α* and IFN-*γ*. The chronic phase leads to a local Th1 response and tissue remodelling with increased deposition of collagen and dermal thickening [41, 42]. We evaluated the effects of SF on systemic immune responses in the lymph nodes and spleen (Supplementary Figures 1(a) and (b)). The lymph nodes play a pivotal role in regulating immune responses and contain many immune cells. Furthermore, the enlargement of lymph nodes is a key indicator of abnormalities in the immune system [43]. In this study, we examined the mRNA expression and production of the proinflammatory cytokines, TNF-*α* and IL-6, which were reduced by SF treatment in NC/Nga mice (Figures 3(a)–3(d)) and in HaCaT keratinocytes (Figures 5(b)–3(e)). Though we had discrepancy between the protein productions and mRNA expressions of cytokines, we would like to assume that it could happen because there are many steps between after the mRNA transcription and before the translation into protein such as posttranscriptional stages and mRNA stability [44]. TNF-*α* and IL-6 are primary proinflammatory cytokines, with a wide range of immune-stimulatory activities. Keratinocytes play a crucial role in the development of inflammatory skin diseases such as AD and psoriasis [45–47].

The production of cytokines is important for the recruitment of inflammatory cells, such as lymphocytes and keratinocytes, and mast cells to the skin in various inflammatory skin diseases [48]. Keratinocytes can amplify the inflammatory response *via* the production of TNF-*α* and IFN-*γ*. Stimulated keratinocytes have been considered crucial sources of proinflammatory cytokines, which influence T lymphocyte differentiation and recruitment of leukocytes to the skin in inflammatory diseases, including AD [49]. Since there is very distinct physiology between the mouse and human skins, we set the NC/Nga mouse model as the *in vitro* model and HaCaT keratinocytes as the *in vivo* model. HaCaT keratinocytes are spontaneously immortalized human keratinocytes maintaining the normal keratinocyte morphology [50]. Therefore, this cell line is frequently used in pharmacological *in vitro* studies on potential skin medicines [51].

Since STATs constitute a family of nuclear proteins that mediate the activation of various inflammatory cytokines, such as interleukins and interferons, inhibition of STAT1 phosphorylation is considered pivotal for the treatment of skin inflammatory disorders [25, 26]. In this study, we investigated the effects of SF on the activation of STAT1 in NC/Nga mice (Figure 4) and in HaCaT keratinocytes (Figure 6).

Although many studies using single phytocompound-based drugs have shown promising results, there is a trend toward the use of the whole plant extracts owing to its potential synergistic effects [52]. In this regard, we decided to investigate the anti-AD effects of SF as a whole plant extract because *Seaweed fulvescens* has many bioactive compounds like chlorophyll which has an anti-inflammatory effect [19] and fucoidan with wound healing and tissue engineering activities [21]. However, there are only a few studies that have focused on the extract. In addition, we will investigate other active compounds responsible for the effects of SF, as well as the underlying mechanistic pathways in a further study. Furthermore, strict clinical trials should be done before stage of new medicine development considering the very distinct physiology of the mouse and human skins.

In conclusion, SF showed anti-AD effects in the Df-induced AD-like mouse model and in TNF-*α* and IFN-*γ* mixture-stimulated HaCaT keratinocytes *via* inhibition of proinflammatory cytokine production and expression. These effects of SF in AD were attributed to the inhibition of the STAT1 signaling pathway. Taken together, the present results suggested that SF might be a potential therapeutic approach for the management of allergic inflammatory diseases, such as AD.

## Figures and Tables

**Table 1:** Sequences of the real-time reverse transcription polymerase chain reaction (RT-PCR) primers.

Gene		
IL-6 (m)	Forward (5′-3′)	TTCCATCCAGTTGCCTTCTTG
	Reverse (5′-3′)	GGGAGTGGTATCCTCTGTGAAGTC
TNF-*α* (m)	Forward (5′-3′)	ATGAGCACAGAAAGCATGAT
	Reverse (5′-3′)	TACAGGCTTGTCACTCGAAT
GAPDH (m)	Forward (5′-3′)	GACGGCCGCATCTTCTTGT
	Reverse (5′-3′)	CACACCGACCTTCACCATTTT
IL-6 (h)	Forward (5′-3′)	ATTCCGGGAACGAAAGAGAA
	Reverse (5′-3′)	TCTTCTCCTGGGGGTACTGG
TNF-*α* (h)	Forward (5′-3′)	GCTGGAGAAGGGTGACCGAC
	Reverse (5′-3′)	GTTCGTCCTCCTCACAGGGC
GAPDH (h)	Forward (5′-3′)	CTCCTCCACCTTTGACGCTG
	Reverse (5′-3′)	CTCTTGTGCTCTTGCTGGGG

## Data Availability

The data used to support the findings of this study are available from the corresponding author upon request.

## Supplementary Materials

Supplementary Materials Supplementary Figure 1: effect of SF on DF-induced systemic immunological abnormalities in NC/Nga mice. The representative image of lymph nodes (a) and spleen (b) from the mice and the weight. Values represent the mean ± SD of three independent experiments. ^###^ *P* < 0.001 versus the control group.
