# Supplementary material for: Anti-Atopic Dermatitis Effect of Seaweed Fulvescens Extract via Inhibiting the STAT1 Pathway
Source: Mediators Inflamm. 2019 Mar 17;2019:3760934. doi: 10.1155/2019/3760934 (PMC6441517; doi:10.1155/2019/3760934)
Supplement: Supplementary Materials — Supplementary Figure 1: effect of SF on DF-induced systemic immunological abnormalities in NC/Nga mice. The representative image of lymph nodes (a) and spleen (b) from the mice and the weight. Values represent the mean ± SD of three independent experiments. ### P < 0.001 versus the control group. [file 3760934.f1.pdf]

Supplementary Materials

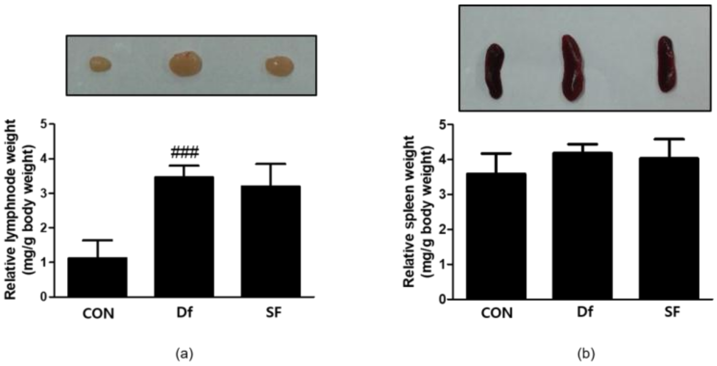

SUPPLEMENTARY FIGURE 1: Effect of SF on DF-induced systemic immunological abnormalities in NC/Nga mice. The representative image of lymph nodes (a) and spleen (b) from the mice and the weight. Values represent the mean ± SD of three independent experiments. $^{###}P<0.001$ versus the control group.
